# Isolated Congenital Membranous Interventricular Septal Aneurysm: A Rare Incidental Finding

**DOI:** 10.7759/cureus.89890

**Published:** 2025-08-12

**Authors:** Hind Hibatouallah, Selma Siagh, Zakia Touati, Mohamed Cherti

**Affiliations:** 1 Cardiology B Department, Ibn Sina Hospital, Rabat, MAR

**Keywords:** case report, incidental finding, interventricular septal defect, membranous interventricular septal aneurysm, right ventricular outflow obstruction

## Abstract

Membranous interventricular septal aneurysm (MVSA) is a rare congenital cardiac anomaly, typically associated with interventricular septal defects. Isolated MVSAs are even more uncommon and often discovered incidentally. While generally asymptomatic, MVSAs carry the potential for significant complications, warranting thorough evaluation and follow-up. We present the case of a 30-year-old physician with no personal or family history of cardiovascular disease, who was referred to our department following the incidental discovery of a cardiac abnormality during a transthoracic echocardiography workshop. The patient was asymptomatic, with a normal physical examination and electrocardiogram. Echocardiography revealed a 21 × 19 mm membranous interventricular septal aneurysm protruding into the right heart chambers, with no evidence of interventricular shunting, right ventricular outflow obstruction, aortic regurgitation, or vegetation. A 24-hour Holter ECG showed no arrhythmias. A conservative management strategy was adopted after a multidisciplinary discussion. The patient was informed of the potential risks, advised to avoid strenuous isometric activities, and scheduled for echocardiographic follow-up every six months during the first year, then annually. At 12-month follow-up, the patient remained asymptomatic with stable findings. This case emphasizes the importance of recognizing and evaluating isolated MVSAs, even in asymptomatic individuals, due to their potential for serious complications.

## Introduction

Membranous interventricular septum aneurysm (MVSA) is a rare congenital cardiac anomaly characterized by a localized outpouching of the membranous portion of the interventricular septum [1]. MVSAs are generally associated with congenital heart diseases, particularly perimembranous ventricular septal defects (VSDs). One echocardiographic series reported this association in 19% of cases [2].

Isolated membranous ventricular septal aneurysms (MVSAs) are rarer, and their true incidence remains unknown, with only a few case reports described in the literature. The clinical presentation of MVSA varies widely, with many patients remaining asymptomatic and diagnosis often made incidentally during imaging studies such as echocardiography [3]. However, MVSAs have been linked to several potential complications, including right ventricular outflow tract obstruction, arrhythmias, conduction disturbances, thromboembolism, and infective endocarditis [4,5].

Given the rarity of this condition and the possible complications, timely and accurate diagnosis is crucial for appropriate management and surveillance. This case report presents an incidentally detected congenital MVSA identified during a routine echocardiography workshop, highlighting the importance of awareness and careful evaluation in congenital heart disease.

## Case presentation

We report the case of a 30-year-old physician referred to our department for evaluation of a cardiac abnormality incidentally identified during a transthoracic echocardiography workshop, where he participated as a volunteer subject. His medical history was unremarkable, and there was no family history of cardiovascular disease or sudden cardiac death. He was entirely asymptomatic, reporting no chest pain, shortness of breath, palpitations, or syncope, and maintained an active lifestyle without any limitations. Physical examination, resting electrocardiogram, and laboratory investigations were all normal.

**Figure 1:**
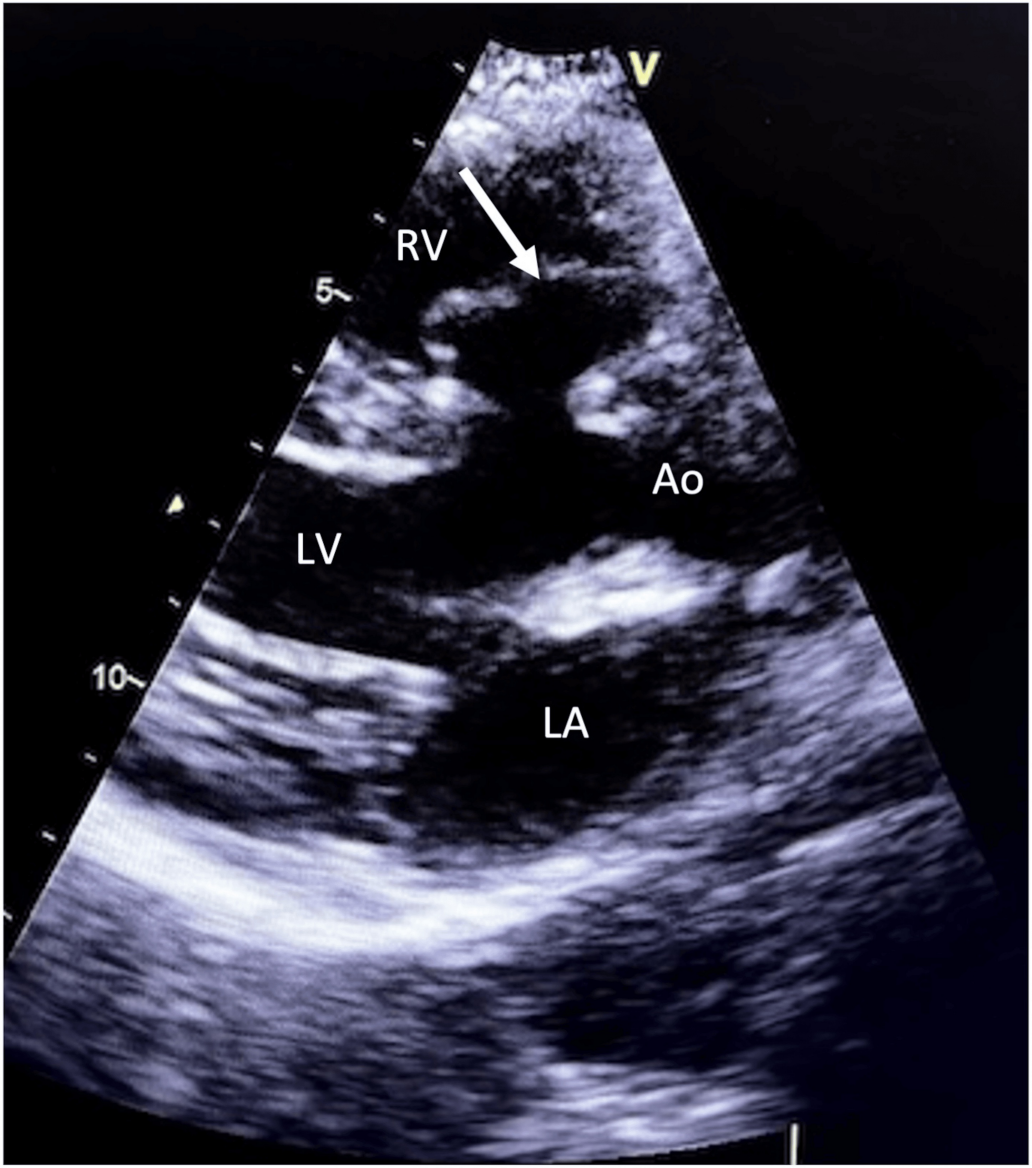
Transthoracic parasternal long-axis view echocardiography showing the membranous ventricular septal aneurysm (arrow). Ao: aorta; LA: left atrium; LV: left ventricle; RV: right ventricle.

**Figure 2:**
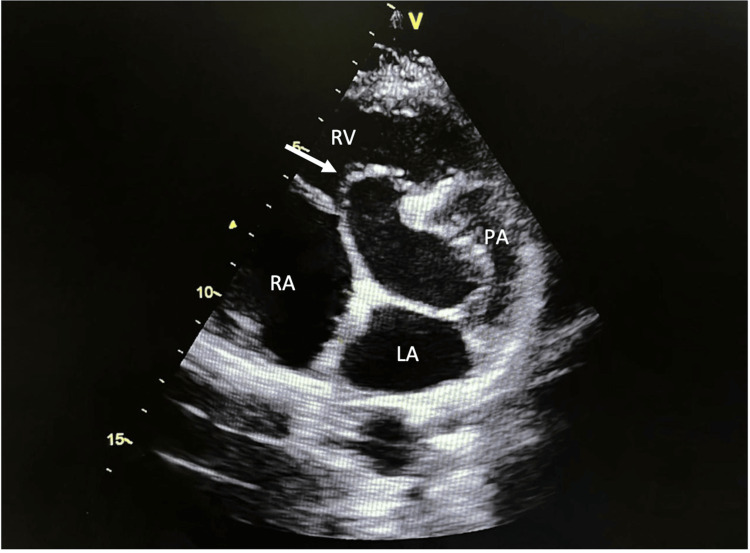
Modified transthoracic parasternal short-axis view showing the membranous interventricular septal aneurysm (arrow). LA: left atrium; PA: pulmonary artery; RA: right atrium; RV: right ventricle.

**Figure 3:**
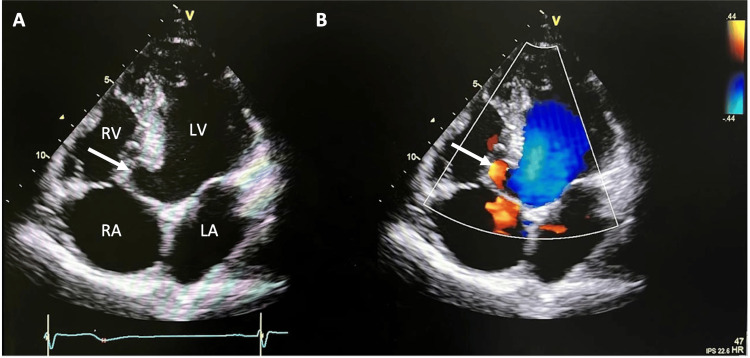
Transthoracic apical four-chamber view echocardiography and Color Doppler. Transthoracic apical four-chamber view echocardiography showing the membranous interventricular septal aneurysm (arrow) measuring 21 × 19 mm (neck width and depth, respectively) (A). Color Doppler overlying the aneurysm revealed no evidence of shunting (B). LA: left atrium; LV: left ventricle; RA: right atrium; RV: right ventricle.

Transthoracic echocardiography (TTE) revealed a membranous interventricular septal aneurysm measuring 21 × 19 mm (width × depth), protruding into the right heart chambers without evidence of right ventricular outflow tract obstruction (Figures 1-3). The aneurysm appeared thin-walled with slight systolic expansion. Color Doppler imaging revealed no evidence of interventricular communication, while spectral Doppler confirmed the absence of significant flow turbulence. Both ventricles were normal in size, with non-hypertrophied walls and preserved systolic and diastolic functions. No valvular abnormalities were detected. Differential diagnoses considered included sinus of Valsalva aneurysm, ventricular diverticulum, and residual VSD; however, echocardiography clearly identified the lesion as a membranous septal aneurysm without shunting or abnormal flow patterns. In view of the adequate diagnostic quality of the images and the absence of associated abnormalities, additional advanced imaging was deemed unnecessary.

The work-up was complemented by a 24-hour Holter ECG, showing no abnormalities. Given the absence of symptoms and complications, the case was discussed in a multidisciplinary meeting, and a conservative management strategy was adopted. The patient was informed of the diagnosis, the potential risks, and advised to avoid high-intensity isometric exercise. Follow-up was initially planned every six months due to the relatively large size of the aneurysm, its thin wall, and the lack of robust data guiding follow-up intervals in such cases. After one year of documented stability, follow-up was safely transitioned to an annual schedule. Infective endocarditis prophylaxis was not recommended, consistent with current guidelines. However, the patient was educated about warning signs and instructed to seek evaluation if needed. At 12-month follow-up, he remained asymptomatic with stable aneurysm dimensions and no new complications on imaging.

## Discussion

The interventricular septum starts developing at the fifth week of gestation [3]. It is composed of four parts: the membranous, inlet, infundibular, and muscular portions. The membranous septum forms through the downward proliferation of tissue from the endocardial cushions, while the muscular interventricular septum develops through the upward proliferation of tissue from the cardiac apex toward the endocardial cushions. The interventricular septum is completed when these two components successfully merge. Due to the lack of myocardial tissue in the membranous area, this region is particularly susceptible to aneurysmal changes when subjected to elevated pressure gradients [6].

MVSAs are usually asymptomatic unless they coexist with other congenital heart anomalies or lead to complications. In such cases, patients can present with a variety of symptoms, including dyspnea, palpitations, signs of heart failure, or syncope [3,7]. Often, MVSAs are discovered incidentally during routine transthoracic or transesophageal echocardiographic examinations performed for unrelated cardiac evaluations [1].

Echocardiography is essential for both the comprehensive evaluation of MVSAs and the early detection of potential complications. It enables accurate assessment of the aneurysm’s size, location, morphology, and its spatial relationship with adjacent cardiac structures. Furthermore, echocardiography is essential for detecting shunt flow, assessing potential hemodynamic repercussions, and verifying the integrity of the adjacent valves. These diagnostic elements are critical for informing clinical decision-making and determining whether additional imaging or therapeutic interventions are necessary [1]. In cases where echocardiographic imaging is suboptimal due to poor acoustic windows, cardiac computed tomography (CT) or magnetic resonance imaging (MRI) can provide superior spatial resolution and contribute significantly to the differential diagnosis. This is particularly important for distinguishing MVSAs from other conditions, such as sinus of Valsalva aneurysms, which typically present as saccular dilatations of the aortic sinus, whereas MVSAs are located immediately beneath the aortic valve [5].

Even when isolated, MVSAs may lead to significant complications, thus warranting structured management. To further elucidate the characteristics of isolated MVSAs, defined as aneurysms occurring without other cardiac anomalies unless they were deemed secondary complications of the aneurysm, we conducted a comprehensive literature review of all reported cases since 1900. This timeframe was chosen because earlier reports often lacked complete clinical and imaging details necessary for standardized analysis. We searched the PubMed database up to July 2025 using keywords including membranous interventricular septal aneurysm, right ventricular outflow obstruction, ventricular tachycardia, atrioventricular block, bundle branch block, thrombus, cerebral embolism, aortic regurgitation, infective endocarditis, rupture, incidental finding, and case report. We also manually reviewed references of included articles. After applying inclusion criteria, we analyzed clinical data from 32 patients with isolated MVSA, including the present case (Table 1).

**Table 1:** Published cases of isolated membranous ventricular septal aneurysm (MVSA) since 1900. 0: none; AV: atrioventricular; BPM: beats per minute; CMR: cardiac magnetic resonance; CT: computed tomography; ECG: electrocardiogram; ICD: implantable cardioverter-defibrillator; LVH: left ventricular hypertrophy; NS: not specified; NYHA: New York Heart Association functional classification; PVC: premature ventricular complex; RBBB: right bundle branch block; RVOT: right ventricular outflow tract; TEE: transesophageal echocardiography; TTE: transthoracic echocardiography; VF: ventricular fibrillation; VT: ventricular tachycardia; YO: years old.

Study	Age, sex	Medical/family history	Symptoms/indication for diagnostic work-up	Cardiovascular examination	ECG	Detection method	Dimensions	Complication	Management	Evolution (at time of publication)
Langenfeld et al., 1990 [8]	42 YO, male	Recurrent syncope (5 years)	Syncope	Normal	Normal	Left ventriculography	NS	VT	Surgical resection	Asymptomatic since surgery
Thomas et al., 1993 [9]	40 YO, female	Recurrent ischemic stroke	Ischemic stroke	Normal	NS	TTE, left ventriculography, cardiac CT	10 x 10 mm	Embolic cerebral infarction	Surgical resection and anticoagulant therapy for 6 months	No events at 10 years
Graffigna et al., 1994 [10]	62 YO female	Recurrent VT	Dyspnea and syncope	NS	NS	TTE, cardiac catheterization	NS	VT	Surgical resection	No events at 88 months
	52 YO, male	Recurrent VT, one episode VF	Palpitations	NS	NS	Cardiac catheterization	NS	VT	Surgical resection after cryoablation around the neck of the aneurysm	No events at 88 months
Sugioka et al., 2002 [11]	63 YO, female	Turner	Dyspnea on exertion	Systolic and diastolic murmurs	Normal	TTE, cardiac catheterization, cardiac CT	50 x 70 mm	None	Surgical resection	NS
Santamaria et al., 2007 [12]	38 YO, female	0	Palpitations, exercise-induced dizziness and fainting	NS	PVCs	TTE, left ventriculography, cardiac CT	24.3 × 32.1 mm	VT	Radiofrequency catheter ablation guided by noncontact mapping system	No events at 6 months
Langer et al., 2007 [13]	33 YO, female	0	Fatigue, palpitations, dyspnea, pre-syncopes and transient bilateral blindness	NS	PVCs	TTE, cardiac CT	3 x 3 x 4 mm	Complete AV block, RVOT obstruction	Surgical resection and two-chamber pacemaker implantation	No events at 24 months
Komatsu et al., 2007 [14]	58 YO, male	0	Asymptomatic	Normal	NS	TTE, cardiac CT	NS	None	None	NS
Love et al., 2009 [15]	42 YO, female	NS	NS	NS	NS	TTE, left ventriculography	NS	RVOT obstruction	Surgical resection	NS
Catakoglu et al., 2009 [16]	52 YO, male	Arterial hypertension, hypercholesterolemia, and smoking	Exertional chest pain for 2 months	NS	NS	TTE, left ventriculography, CMR	NS	Moderate aortic regurgitation	Surgical resection and aortic valve replacement	No events at 8 months
Jang et al., 2010 [17]	43 YO, female	Arterial hypertension, smoking	Dyspnea and chest discomfort	NS	Complete AV block	TTE, left ventriculography, cardiac CT	NS	Complete AV block	Two-chamber pacemaker implantation + monitoring of the aneurysm	NS
Akashi et al., 2011 [18]	71 YO, female	0	Syncope	NS	VT	Left ventriculography, cardiac CT	15×13 mm	VT	Two ablations with recurrent VT; then ICD implantation; monitoring of the aneurysm	Event-free since ICD implantation
Choi et al., 2011 [19]	49 YO, male	0	Chest pain	NS	LVH	Cardiac CT	19 x 13 mm	NS	NS	NS
	60 YO, female	Arterial hypertension, hyperlipidemia	Dyspnea	NS	Incomplete RBBB	Cardiac CT	16 x 10 mm	NS	NS	NS
	48 YO, male	Arterial hypertension	NS	NS	LVH	Cardiac CT	22 x 14 mm	NS	NS	NS
	68 YO, female	Diabetes mellitus	NS	NS	First degree AV block, incomplete RBBB	Cardiac CT	19 x 12 mm	NS	NS	NS
	61 YO, female	Arterial hypertension, hyperlipidemia	Palpitation	NS	Normal	TTE, cardiac CT	10 x 5 mm	NS	NS	NS
Naidu et al., 2012 [1]	18 YO, female	0	Asymptomatic	Bradycardia (45 bpm), systolic murmur		TTE, TEE, cardiac CT	12 x 9 mm	0	Routine monitoring, continued competitive athletics allowed	NS
Stendahl et al., 2014 [20]	41 YO, male	0	Ischemic stroke	NS	NS	TTE, cardiac CT	NS	Thromboembolism	Close clinical follow-up	No events at 4 months
Di Cesare et al., 2014 [21]	62 YO, female	0	Clinic suspicion of acute myocarditis	NS	NS	CMR	20 × 15 mm	0	Strictly CMR follow-up	NS
Guerrero Marquez et al., 2016 [22]	46 YO, female	Smoking, recurrent syncope (12 years)	Syncope	Normal	Paroxysmal complete AV block	TTE, TEE	11 mm	Paroxysmal complete AV block	Dual-chamber pacing device implantation	No events during subsequent follow-up
Abdul Jabbar et al., 2017 [23]	65 YO, male	NS	Chest pain and dyspnea on exertion	NS	NS	TTE, TEE with agitated saline enhancement	14 x 12 x 22 mm	0	Close follow-up	NS
	65 YO, male	Colon cancer and rheumatoid arthritis	Recurrent nonsustained VT	NS	NS	TTE	NS	Thrombus, VT	The patient declined surgery	NS
	54 YO, male	NS	Chest pain	NS	NS	Cardiac angiography, cardiac CT	NS	0	Close follow-up	NS
Ivanitskaya et al., 2018 [24]	0 YO, female	Family history of trisomy 18 (sibling)	Prenatal suspicion of atrioventricular septal defect	NS	NS	TEE	3 mm	0	Close follow-up	3 months follow-up showed no septal anomaly
Theodoropoulos et al., 2017 [25]	34 YO, female	Community-acquired pneumonia requiring hospitalization	Clinical suspicion of pericarditis (probably related to the pneumonia)	Systolic murmur	Normal	TTE, TEE	17 x 18 mm	0	Follow-up	No events at 18 months
Wang et al., 2018 [26]	34 YO, female	NS	NYHA class II dyspnea	NS	NS	TEE	8 x 8 mm	NS	NS	NS
Dell’Angela et al., 2021 [27]	62 YO, male	Arterial hypertension, smoking and alcohol abuse	Syncope	NS	NS	TTE, cardiac CT, CMR	21.2 × 19.3 × 19.9 mm	Mild aortic regurgitation	Clinical and echocardiographic follow‐up at 6 months, then every 9-12 months	No events at 6 months
Assaf et al., 2021 [5]	42 YO, male	Hyperlipidemia, smoking	Palpitations	Normal	Left anterior hemiblock	TEE, cardiac CT	13×17 mm	0	Follow-up every 3 to 5 years	NS
Kumar et al., 2023 [2]	54 YO, male	Arterial hypertension, smoking	Chest pain, dyspnea, and nausea	Diastolic murmur at apex	Normal	TEE, cardiac CT, CMR	30 x 35 x 50 mm	0	Surgical resection (given the large size of the aneurysm and its location)	NS
Kim et al., 2024 [28]	54 YO, male	History of ischemic stroke 6 years previously	Ischemic stroke	NS	Normal	TTE, cardiac CT	NS	Embolic cerebral infarction	Life-long warfarin; surgery if embolism recurs	Thrombus nearly resolved after 1 month warfarin
Present case	30 YO male	0	Asymptomatic	Normal	Normal	TTE	21 x 19 mm	0	Follow‐up at 6 months, then annually	No events at 12 months

Among the complications reported, right ventricular outflow tract (RVOT) obstruction is caused by systolic bulging of the aneurysm into the outflow tract due to left ventricular pressure. This mechanical interference can lead to subpulmonic stenosis [4,29]. Langer et al. described a 33-year-old woman who presented with fatigue, palpitations, and presyncope [13]. A 3 × 3 × 4 mm MVSA caused RVOT obstruction and complete atrioventricular (AV) block, leading to surgical resection and pacemaker implantation, with a favorable outcome [14]. In fact, conduction disturbances are possible due to the aneurysm’s close proximity to the His bundle and AV node. These may result in complete AV block or bundle branch block, likely from compression or fibrosis of conduction tissue [23].

Furthermore, MVSAs have been implicated in the development of ventricular tachyarrhythmias. The aneurysmal structure may serve as a substrate for macro-reentrant circuits, resulting in sustained ventricular tachycardia (VT). Langenfeld et al. and Graffigna et al. each reported cases of VT successfully treated with surgical resection, while Santamaria et al. opted for catheter ablation [8,10,12]. In cases of recurrence, implantable cardioverter-defibrillator (ICD) therapy has also been reported, such as in the case by Akashi et al. [18].

In addition to arrhythmic risks, the abnormal wall motion within the aneurysmal segment can promote thrombus formation and a subsequent increased risk of systemic embolization. Thomas et al. reported the case of a 40-year-old woman with recurrent cerebral emboli, in whom a 10 × 10 mm MVSA was identified and successfully resected, with no recurrence over 10 years of follow-up [9].

Isolated MVSAs have also been associated with aortic valve regurgitation. Catakoglu et al. and Dell’Angela et al. each described mild to moderate aortic regurgitation in such contexts [16,27]. While a direct causal relationship remains unconfirmed, it is plausible that the proximity of the aneurysm to the posterior and right aortic cusps may compromise aortic valve integrity and function.

Finally, high-velocity blood flow jets near or within the aneurysm may facilitate sterile thrombus formation, potentially serving as a nidus for infective endocarditis. Moreover, the fragile structure of the membranous septum raises theoretical concerns regarding aneurysm rupture. Although these complications remain hypothetical in the absence of large-scale longitudinal studies, their potential clinical implications warrant careful consideration [3].

Despite these potential risks, there are currently no established guidelines specifically addressing the management of isolated MVSAs. This reflects the rarity of the condition and the absence of expert consensus [2]. In the majority of uncomplicated cases, a conservative approach with regular follow-up was adopted. However, follow-up intervals varied considerably across reports. For instance, Assaf et al. recommended clinical and imaging follow-up every three to five years, whereas Dell’Angela et al. proposed follow-up at six months, then every nine to 12 months [5,27]. We believe that aneurysm diameter and the indexed cross-sectional area should be considered when determining individualized surveillance frequency [5,27].

In our case, a conservative management strategy was adopted, with annual outpatient follow-up. The patient was thoroughly informed about the anomaly and educated on warning signs such as new-onset dyspnea, chest pain, palpitations, or syncope, with instructions to seek prompt medical attention if such symptoms occur. Given the incidental and isolated nature of the aneurysm, and the absence of syndromic features or a family history of cardiac disease, neither genetic counseling nor familial screening was considered necessary.

Surgical intervention is generally reserved for cases involving associated cardiac anomalies, onset of complications, or significant hemodynamic compromise. However, surgery carries inherent risks, particularly due to the close anatomical relationship between the membranous septum, the septal leaflet of the tricuspid valve, and the conduction system. Reported surgical complications include tricuspid regurgitation and complete AV block [5].

## Conclusions

Although typically hemodynamically insignificant, isolated MVSAs carry potential risks that warrant structured follow-up and patient education. Diagnosis should prompt a comprehensive cardiac evaluation to exclude associated anomalies and detect early complications. In most cases, including the one reported here, conservative management with regular outpatient follow-up is appropriate, as the overall prognosis is generally favorable in the absence of complications and hemodynamic disturbance. However, the unpredictable nature of potential complications justifies ongoing surveillance. This case underscores the clinical relevance of incidental cardiac findings, which may have broader implications than initially assumed. Larger prospective case series and registries are needed to better define the natural history, optimal management strategies, and long-term prognosis of isolated MVSAs.
